# ﻿A revision of the genus *Eurymesosa* Breuning, 1938 (Cerambycidae, Lamiinae, Mesosini)

**DOI:** 10.3897/zookeys.1193.115513

**Published:** 2024-03-05

**Authors:** Gui-Qiang Huang, Ling-Rui Xu, Xian Zhou, Gui-Mei Zhang

**Affiliations:** 1 School of Biological Science and Technology, Liupanshui Normal University, Liupanshui 553004, Guizhou, China Liupanshui Normal University Liupanshui China

**Keywords:** Longhorn beetles, Oriental region, redescription, resurrection, synonymy, taxonomy

## Abstract

A taxonomic revision and redescription of the genus *Eurymesosa* Breuning, 1938 are presented, including a key to species. Three of the five currently accepted species are considered valid: *Eurymesosaventralis* (Pascoe, 1865), *Eurymesosaallapsa* (Pascoe, 1866) and *Eurymesosaziranzhiyi* Yamasako & Lin, 2016. Three junior synonyms are proposed for *E.ventralis*: *Eurymesosaalbostictica* Breuning, 1962, **syn. nov.**, *Eurymesosaaffinis* Breuning, 1970, **syn. nov.**, and *Eurymesosamultinigromaculata* Breuning, 1974, **syn. nov.** Additionally, *E.allapsa* (Pascoe, 1866) is resurrected from synonyms of *E.ventralis*. Females of *E.allapsa* and *E.ziranzhiyi* Yamasako & Lin, 2016 are described for the first time.

## ﻿Introduction

The genus *Eurymesosa* presently consists of five species distributed in East Asia and Southeast Asia (Tavakilian and Chevillotte 2023). It was established by Breuning (1938) within the tribe Mesosini Mulsant, 1839 based on the species *Ereisventralis* Pascoe, 1865. Subsequently, *Eurymesosaalbostictica* Breuning, 1962 and *Eurymesosaaffinis* Breuning, 1970 were described from Laos (Breuning 1962, 1970), *Eurymesosamultinigromaculata* Breuning, 1974 was described from Cambodia (Breuning 1974) and *Eurymesosaziranzhiyi* Yamasako & Lin, 2016 (only male) was described from China (Yamasako and Lin 2016).

We found that the taxonomic status of *E.albostictica*, *E.affinis*, *E.multinigromaculata* and *Mesosaallapsa* Pascoe, 1866 [currently a junior synonym of *Eurymesosaventralis* (Pascoe, 1865)] are doubtful. Moreover, in some cases the sex of the type specimens was not specified in the original description. Therefore, this paper aims to revise and redescribe the genus *Eurymesosa*.

## ﻿Materials and methods

The specimens examined are deposited in following institutional and private collections:

**BMNH** The Natural History Museum, London, United Kingdom

**CDJH** Collection Daniel J. Heffern, Houston, Texas, United States

**CFV** Collection Francesco Vitali, Luxembourg, Grand-Duchy of Luxembourg

**CZJL** Collection Zi-Jun Liu, Xi’an, Shaanxi, China

**IZCAS** Insect collection of the Institute of Zoology, Chinese Academy of Sciences, Beijing, China

**LPSNU** School of Biological Science and Technology, Liupanshui Normal University, Liupanshui, Guizhou, China

**MNHN** Muséum National d’Histoire Naturelle, Paris, France

**YZU** The Insect Collection, College of Agriculture, Yangtze University, Jingzhou, Hubei, China

The methods of taking photographs (Figs 4A–D, 5A–D) followed Huang et al. (2020). The photographs were taken with a Canon EOS 5DSR camera equipped with a Canon AF 100 mm macro lens and connected to the software Helicon Remote (Ver. 3.9.7 W); top and bottom focus of the specimens were chosen by adjusting the focus of the lens using Helicon Remote; the shoot was commenced to obtain images at different depths of focus; and finally, images were stacked into a single high resolution image with the software Helicon Focus (Ver. 6.7.1). The copyrights of other photographs were added to legend of corresponding figures. All photographs and figures were produced using Photoshop CS5 software.

## ﻿Taxonomy

### ﻿ Eurymesosa

Taxon classificationAnimaliaColeopteraCerambycidae

Breuning, 1938

4ABC96FF-4E3D-5FCE-ADA2-948BBD2FA0B0

Eurymesosa Breuning, 1938: 366 (key), 391 (original description); Breuning 1959: 49 (catalogue); Rondon and Breuning 1970: 319 (catalogue); Yamasako and Lin 2016: 194 (diagnosis, distribution); Lin and Yang 2019: 331 (catalogue); Danilevsky 2020: 390 (catalogue).

#### Type species.

*Ereisventralis* Pascoe, 1865.

#### Redescription.

Breuning (1938) described detailed characters in his original description of *Eurymesosa*, but we found it is necessary to improve the description of this genus after examining types of all species and additional material. Thus, we redescribe *Eurymesosa* based on the original description provided by Breuning.

Body elongated oval and robust. Head with single narrow and medial shallow sulcus extending from base of frons to posterior of vertex. Eyes coarsely faceted. Antennae moderately thin, sparsely fringed with long dark brown pubescence beneath, antennae more than 1/2 length of body in males, about 1/4 longer than body in females, apical cicatrix of antennal scape opened, 3^rd^ antennomere significantly longer than scape and 4^th^ antennomere respectively. Pronotum transverse and slightly rounded laterally, with three irregular calluses (two located at sides of center and one near basal middle), with single anterior transverse groove (middle part nearly missing) and single posterior transverse groove; disc sparsely covered with short white setae. Prosternal process narrow and distinctly lower than procoxae; procoxal cavity closed posteriorly. Scutellum linguiform. Elytra elongated, distinctly wider than pronotum, expanded in middle, widely rounded at apex, with two large, oblique bumps behind middle of base; disc sparsely with coarse granules at base and punctation (punctation slightly coarse at about basal 2/3 of elytra and slightly fine at about apical 1/3 of elytra); each elytron sparsely covered with short white setae; with single sub-rounded or sub-oval dark brown haired spot on above bump, single irregular dark brown haired spot behind humeri and close to margin, single sub-rounded dark brown haired spot before middle, several dark brown haired spots behind middle (number and shape of maculae are different in different species), and with several patchy dark brown maculae near apex. Mesosternal process with single tubercle in center, midcoxal cavity open to epimeron externally. Femora strongly claviform, mid-tibiae without groove.

#### Differential diagnosis.

Based on the descriptions of the genera *Eurymesosa* and *Mesosa* Latreille, 1829 provided by Breuning (1938), we found that *Eurymesosa* is similar to *Mesosa* in its elongated oval body, the antennae thin and fringed beneath, the apical cicatrix of antennal scape opened, the 3^rd^ antennomere significantly longer than scape, the elytra widely rounded at apex, the prosternal process lower than procoxae, and the mid-tibiae without a groove. However, *Eurymesosa* differs from *Mesosa* in having the eyes strongly reniform (upper lobe and lower lobe of eyes subdivided in *Mesosa*), the elytra with two large, oblique bumps behind the middle of the base (without two large, oblique bumps behind middle of base in *Mesosa*), the mesosternal process with a single tubercle in the middle (without tubercles in middle in *Mesosa*).

#### Distribution.

Cambodia, China, Indonesia (parts of Borneo), Laos, Malaysia (Peninsular Malayasia, parts of Borneo), Vietnam.

### ﻿ Eurymesosa allapsa

Taxon classificationAnimaliaColeopteraCerambycidae

(Pascoe, 1866), stat. resurr.

1D4966D5-9C18-513A-A17C-6FE79F1B8049

Fig. 1A–I

Mesosa allapsa Pascoe, 1866: 231 (type locality: “Penang, Malaysia”).
Eurymesosa ventralis m. allapsa: Breuning 1938: 391; Breuning 1959: 49 (catalogue).

#### Type material examined.

**Figure 1.**
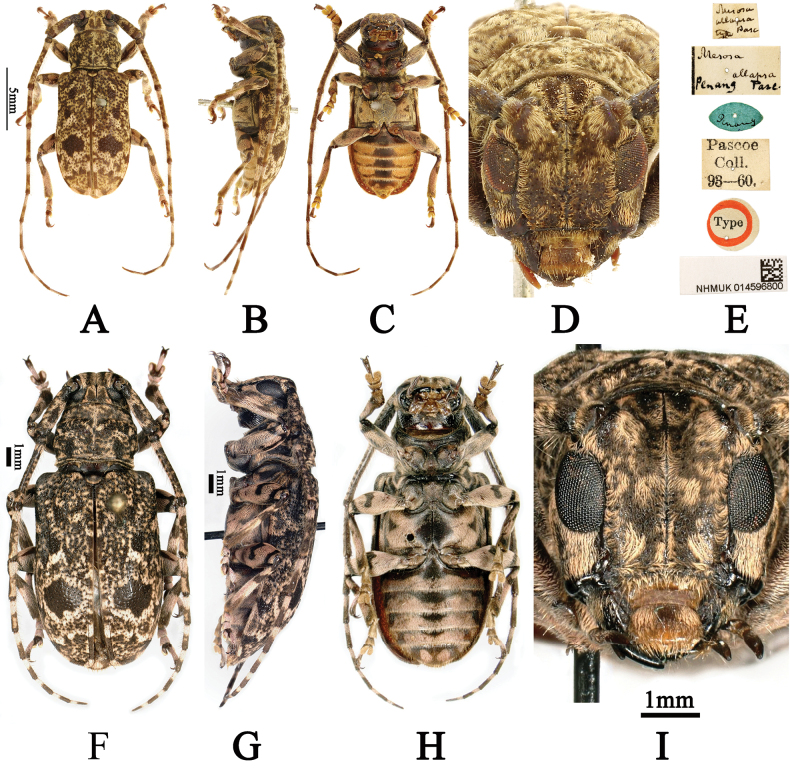
**A–I***Eurymesosaallapsa***A–E***Mesosaallapsa*, holotype **A** male, dorsal habitus **B** male, lateral habitus **C** male, ventral habitus **D** male, frontal view **E** labels (photographs **A–E** were taken by Guang-Lin Xie) **F–I***Eurymesosaallapsa*, female **F** dorsal habitus **G** lateral habitus **H** ventral habitus **I** frontal view (photographs **F–I** were taken by Francesco Vitali).

***Holotype***, ♂ (BMNH), *Mesosaallapsa* Typ Pasc (handwritten with black ink on a rectangular white label) / *Mesosaallapsa* Penang Pasc. (handwritten with black ink on a rectangular white label with a longitudinal black line at left side) / Penang (handwritten with black ink on an olive-green label) / Pascoe Coll. 93–60. (printed with black ink on a rectangular white label) / Type (printed with black ink on a circular white label with circular red borders) / NHMUK 014596800 plus a QR (quick response) code (printed with black ink on a rectangular white label); examined from five photographs (Fig. 1A–E).

#### Additional materials examined.

1 ♀ (CFV), Mt. Bawang, Kalimantan, Borneo, Indonesia, II.2018, leg. local collector; examined from four photographs (Fig. 1F–I). 3 ♀♀ (CDJH), all from Sabah (Crocker Range, 18.III.1999; Mt. Trus-Madi, 14.IV.2001; Ranau, 17.II.2005), Borneo, Malaysia, leg. local collectors. 1 ♂ (CDJH), Tawau, Sabah, Borneo, Malaysia, 1.V.2016, leg. local collector.

#### Description of female.

Similar to male, but with the body length: 14.0–15.4 mm (4 specimens). One of the specimens (Fig. 1F–I), body length: 15.4 mm, antennae 1.28 times as long as body, length (mm) of each antennomere: scape = 3.4, pedicel= 0.4, III = 3.8, IV= 2.7, V = 2.0, VI = 1.5, VII = 1.4, VIII = 1.2, IX = 1.2, X= 1.1, X I = 1.0; elytra 1.6 times as long as wide.

#### Comments.

**Figure 2.**
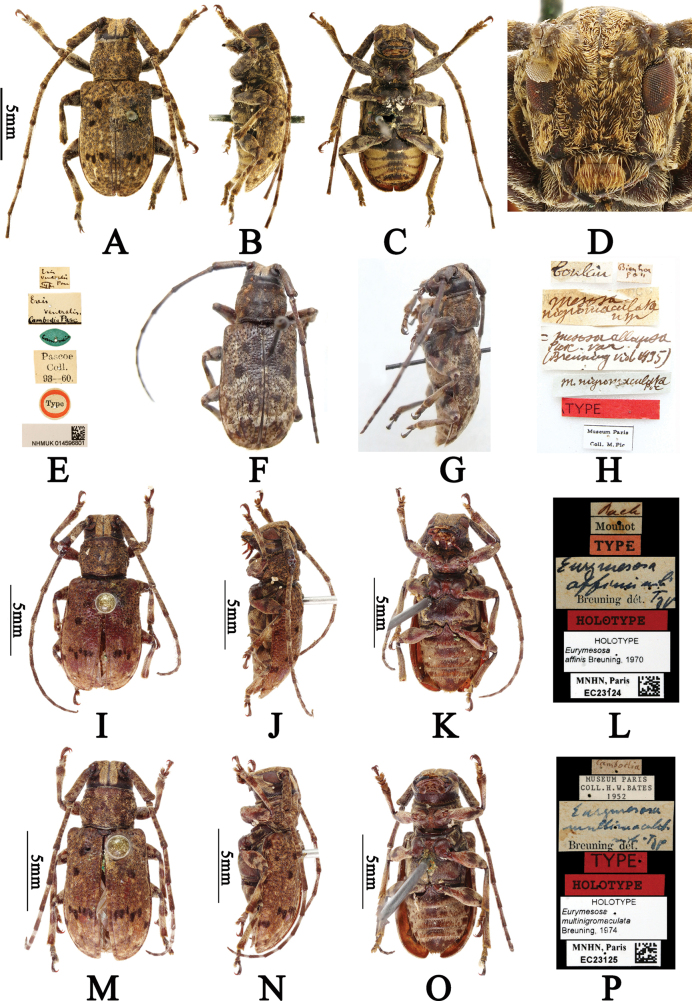
**A–P***Eurymesosaventralis***A–E***Ereisventralis*, holotype **A** male, dorsal habitus **B** male, lateral habitus **C** male, ventral habitus **D** male, frontal view **E** labels (photographs **A–E** were taken by Guang-Lin Xie) **F–H***Mesosanigromaculata*, holotype **F** female, dorsal habitus **G** female, lateral habitus **H** labels (photographs **F–H** were taken by Xavier Gouverneur) **I–L***Eurymesosaaffinis*, holotype **I** female, dorsal habitus **J** female, lateral habitus **K** female, ventral habitus **L** labels **M–P***Eurymesosamultinigromaculata*, holotype **M** female, dorsal habitus **N** female, lateral habitus **O** female, ventral habitus **P** labels (photographs **I–P** were taken by Christophe Rivier).

After exposing the lateral lobes of the tegmen (Fig. 1C), it was possible to confirm that the holotype of *M.allapsa* is a male. Breuning (1938) treated *M.allapsa* as an infraspecific variation or morph of *E.ventralis* based on the character “The two dark brown postmedian disc spots on each elytron are joined by a single larger spot”. After comparing the holotypes of the above two species (Figs 1A–D, 2A–D), we found that *M.allapsa* can be clearly distinguished from *E.ventralis* by the following characters: the pubescent bands on the vertex are brown with light pink border (pubescent bands on vertex are yellowish brown for *E.ventralis*), each elytron covered with patchy dark brown maculae in basal half, with a single large irregular black spot behind middle, with patchy dark brown maculae in middle and near apical 1/4 (each elytron covered with patchy yellowish-brown maculae in basal half, with several small irregular black spots behind middle, with patchy yellowish-brown maculae in middle and near apical 1/4 for *E.ventralis*), femora, apical 2/3 of tibiae and dorsum of two basal joints and last joint of tarsi covered with short light pink pubescence (femora, apical 2/3 of tibiae and dorsum of two basal joints and last joint of tarsi covered with short yellowish-brown pubescence for *E.ventralis*). We thus resurrect *M.allapsa* and decide to keep it in the genus *Eurymesosa*.

#### Distribution.

Malaysia (Penang, Sabah), Indonesia (Kalimantan).

### ﻿ Eurymesosa ventralis

Taxon classificationAnimaliaColeopteraCerambycidae

(Pascoe, 1865)

ED66246D-E3AD-5775-9FAE-7389DB3BBC95

Fig. 2A–P

Ereis ventralis Pascoe, 1865: 105 (type locality: “Cambodia”).
Eurymesosa ventralis : Breuning 1938: 391 (redescription); Breuning 1959: 49 (catalogue).
Mesosa nigromaculata Pic, 1932: 26 (type locality: “Tonkin, Vietnam”).
Eurymesosa ventralis m. nigromaculata: Breuning 1938: 391; Breuning 1959: 49 (catalogue).
Eurymesosa albostictica Breuning, 1962:15 (type locality: “Vientiane, Laos”); Rondon and Breuning 1970: 319, fig. 1b. syn. nov.
Eurymesosa affinis Breuning, 1970: 363 (type locality: “Laos”). syn. nov.
Eurymesosa multinigromaculata Breuning, 1974: 72 (type locality: “Cambodia”). syn. nov.

#### Type materials examined.

***Ereisventralis* Pascoe, 1865: *holotype***, ♂ (BMNH), *Ereisventralis* Typ Pasc (handwritten with black ink on a rectangular white label) / *Ereisventralis* Cambodia Pasc (handwritten with black ink on a rectangular white label with a black line under “Cambodia Pasc”) / Cambodia (handwritten with black ink on a olive-green label) / Pascoe Coll. 93–60. (printed with black ink on a rectangular white label) / Type (printed with black ink on a circular white label with circular red borders) / NHMUK 014596801 plus a QR (quick response) code (printed with black ink on a rectangular white label); examined from five photographs (Fig. 2A–E). ***Mesosanigromaculata* Pic, 1932: *holotype***, ♀ (MNHN), Tonkin (handwritten with black ink on a rectangular white label) / Bien hoa (handwritten with black ink on a rectangular white label) / *Mesosanigromaculata* n sp (handwritten with black ink on a rectangular white label) / *Mesosaallapsa* Pasc. var. (Breuning vid 1935) (handwritten with black ink on a rectangular white label) / *M.nigromaculata* Pic (handwritten with black ink on a rectangular white label) / TYPE (printed with black ink on a rectangular red label) / Museum Paris Coll. M. Pic (printed with black ink on a rectangular white label with black borders); examined from three photographs (Fig. 2F–H). ***Eurymesosaaffinis* Breuning, 1970: *holotype***, ♀ (MNHN), Pach Mouhot (“Pach” handwritten and “Mouhot” printed with black ink on a rectangular white label with a transversal back line in middle) / TYPE (printed with black ink on a rectangular red label) / *Eurymesosaaffinis* mihi Typ Breuning dét. (“*Eurymesosaaffinis* mihi Typ” handwritten and “Breuning dét.” printed with black ink on a rectangular white label) / HOLOTYPE (printed with black ink on a rectangular red label) / HOLOTYPE *Eurymesosaaffinis* Breuning, 1970 (printed with black ink on a rectangular white label) \ MNHN, Paris EC23124 plus a QR (quick response) code (printed with black ink on a rectangular white label); examined from four photographs (Fig. 2I–L). ***Eurymesosamultinigromaculata* Breuning, 1974: *holotype***, ♀ (MNHN), Cambodia (handwritten with black ink on a rectangular white label) / MUSEUM PARIS COLL. H.W. BATES 1952 (printed with black ink on a rectangular white label) / *Eurymesosamultinigromaculata* mihi Typ Breuning dét. (“*Eurymesosamultinigromaculata* mihi Typ” handwritten and “Breuning dét.” printed with black ink on a rectangular white label) / TYPE (printed with black ink on a rectangular red label) / HOLOTYPE (printed with black ink on a rectangular red label) / HOLOTYPE *Eurymesosamultinigromaculata* Breuning, 1974 (printed with black ink on a rectangular white label) / MNHN, Paris EC23125 plus a QR (quick response) code (printed with black ink on a rectangular white label); examined from four photographs (Fig. 2M–P).

#### Comments.

After comparing the holotypes of *E.ventralis* (Fig. 2A–D), *E.albostictica* (the holotype photograph is available at: http://bezbycids.com/byciddb/wdetails.asp?id=31562&w=o), *E.affinis* (Fig. 2I–K) and *E.multinigromaculata* (Fig. 2M–O), we found they were identical except for gender and body color. Pascoe (1865) did not mention the body color of *Ereisventralis* in the original description, while Breuning (1938) transferred *E.ventralis* to *Eurymesosa* and stated that its body color was dark brown; the body color of *E.albostictica* was dark brown in the original description (Breuning 1962). The body color of *E.affinis* and *E.multinigromaculata* are reddish brown, but Breuning (1974) described the body color of *E.multinigromaculata* as dark brown in the original description. Perhaps the body color of *E.multinigromaculata* had faded to reddish brown and similarly for *E.affinis*.

There is no information in the literature regarding the sex of the holotypes of *E.ventralis*, *M.nigromaculata*, *E.affinis* and *E.multinigromaculata*, but we could confirm that the holotype of *E.ventralis* is a male, and the holotypes of *M.nigromaculata*, *E.affinis* and *E.multinigromaculata* are females, based on the description of *Eurymesosa* provided by Breuning (1938) and referring to other species (antennae are about 1/4 longer than body in females).

Breuning (1938) provided the following localities for *E.ventralis* (currently *E.ventralis* + *E.allapsa*): “Cambodge; Presqu’ île de Malacca: Penang (PASCOE); Tonkin: Hoa-Binh (collection Pic); Java; Bornéo: Sandakan (Musée de Dresde)”. We verified the localities of *E.ventralis* across Cambodia, Laos and Vietnam through the examined material (Fig. 2A–P). However, we could not confirm whether the Java locality mentioned by Breuning (1938) pertains to *E.allapsa* or *E.ventralis*; hence, we omitted the Java locality from the records of *E.ventralis*.

#### Distribution.

Cambodia, Laos (Pachbon, Vientane), Vietnam (Hoa-Binh).

### ﻿ Eurymesosa ziranzhiyi

Taxon classificationAnimaliaColeopteraCerambycidae

Yamasako & Lin, 2016

331B7E2D-C23D-5F67-87EE-DFE1CC0C1A26

**Figure 3.**
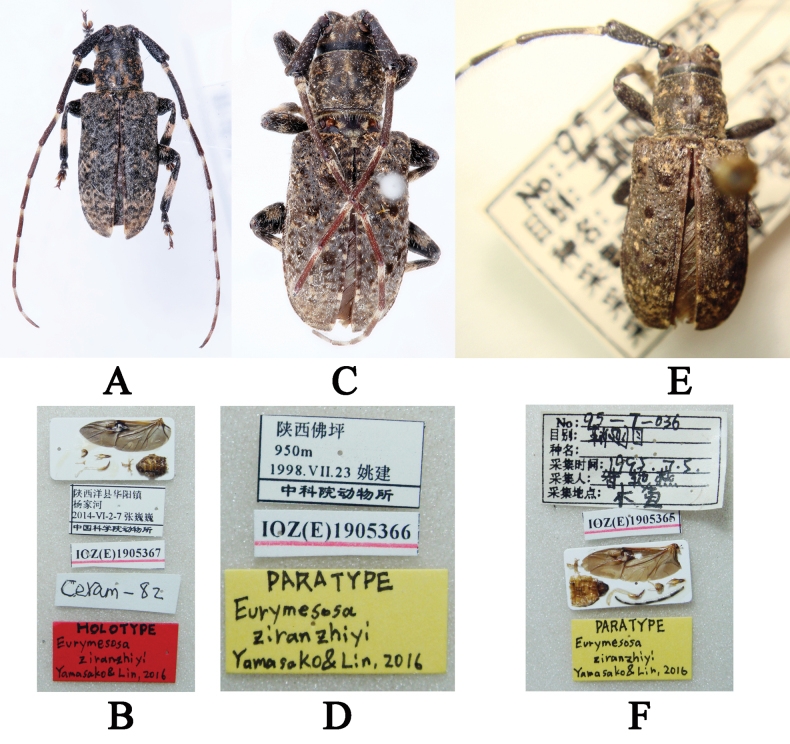
**A–F***Eurymesosaziranzhiyi***A** holotype male, dorsal habitus **B** holotype labels **C** paratype male, dorsal habitus **D** paratype labels **E** paratype male, dorsal habitus **F** paratype labels (all photographs were taken by Mei-Ying Lin).

**Figure 4.**
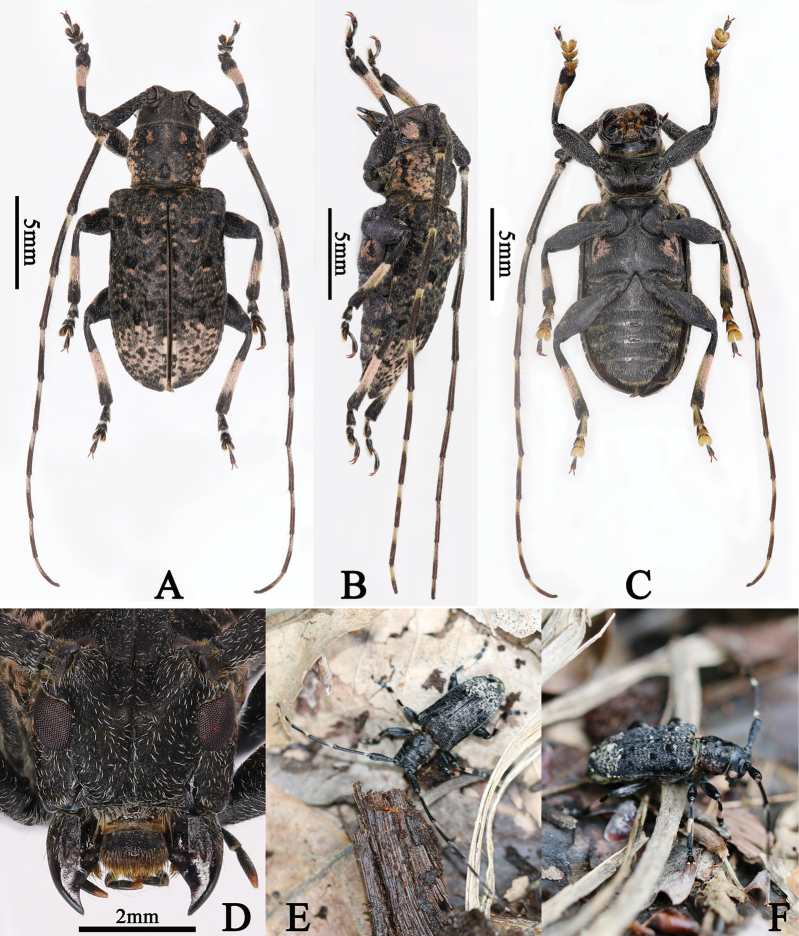
**A–F***Eurymesosaziranzhiyi*, male **A** dorsal habitus **B** lateral habitus **C** ventral habitus **D** frontal view (photographs **A–D** were taken by Xian Zhou) **E–F** live adult **E** dorsal habitus **F** lateral habitus (photographs **E–F** were taken by Mao Ye).

**Figure 5.**
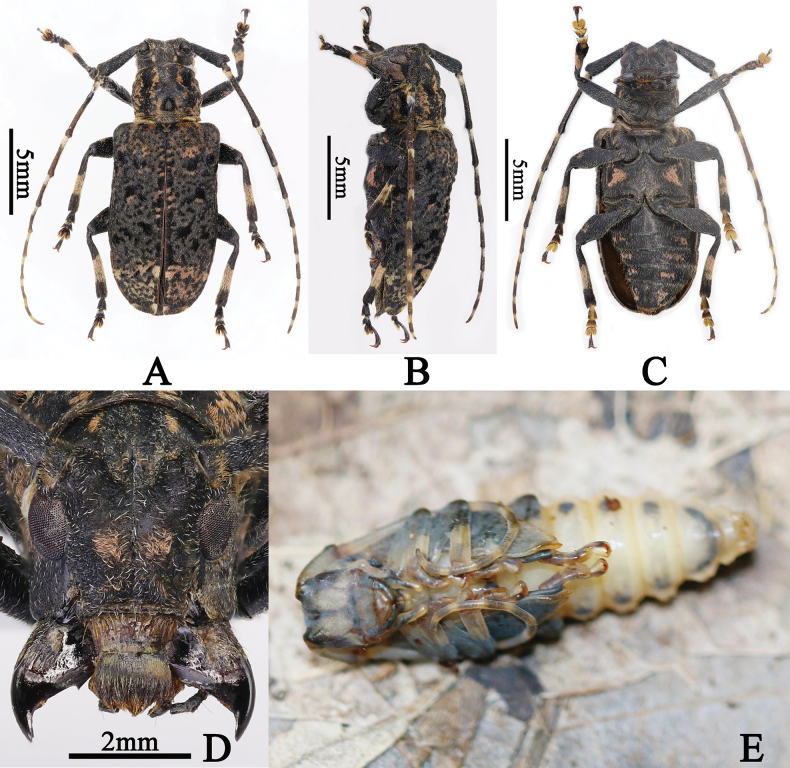
**A–E***Eurymesosaziranzhiyi*, female **A** dorsal habitus **B** lateral habitus **C** ventral habitus **D** frontal view (photographs **A–D** were taken by Xian Zhou) **E** living pupa (photograph **E** was taken by Mao Ye).

Figs 3A–F , 4A–F , 5A–E

Eurymesosa ziranzhiyi Yamasako & Lin, 2016: 194 (type locality: “Yangjiahe, Huayangzhen, Yangxian, Shaanxi, China”), figs 1–3 (holotype, male), 4–10 (holotype, male genitalia); Lin and Yang 2019: 331 (catalogue); Danilevsky 2020: 390 (catalogue).

#### Type materials examined.

***Holotype***, ♂ (IZCAS): left hind wing, male terminalia, metendosternite and abdomen are affixed with glue onto a rectangular white label / 陕西洋县华阳镇杨家河 2014-VI-2-7 张巍巍 中国科学院动物所 (printed with black ink on a rectangular white label with black borders) / IOZ(E)1905367 (printed in black ink on a rectangular white label with a red underline) / Ceram-82 (handwritten with black ink on a rectangular white label) / HOLOTYPE *Eurymesosaziranzhiyi* Yamasako & Lin, 2016 (handwritten with black ink on a rectangular red label); examined from two photographs (Fig. 3A–B). ***Paratype***, 1♂ (IZCAS): 陕西佛坪 950m 1998.VII.23 姚建 中科院动物所 (printed with black ink on a rectangular white label with black borders) / IOZ(E)1905366 (printed in black ink on a rectangular white label with a red underline) / PARATYPE *Eurymesosaziranzhiyi* Yamasako & Lin, 2016 (handwritten with black ink on a rectangular yellow label); examined from two photographs (Fig. 3C–D). ***Paratype***, 1♂ (IZCAS): No:95–7–036 目别:鞘翅目 种名: 采集时间:1993.3.5 采集人:昝艳燕 采集地点:木鱼 (“No:目别:种名:采集时间:采集人:采集地点:” with a black underline printed and “95–7–036 鞘翅 1993.3.5 昝艳燕 木鱼” handwritten with black ink on a rectangular white label) / IOZ(E)1905365 (printed in black ink on a rectangular white label with a red underline) / metendosternite, left hind wind, abdomen, male terminalia and antennomeres VI–XI were pasted with glue on a rectangular white label / PARATYPE *Eurymesosaziranzhiyi* Yamasako & Lin, 2016 (handwritten with black ink on a rectangular yellow label); examined from two photographs (Fig. 3E–F).

#### Additional materials examined.

1♂ (LPSNU, fig. 4A–F), 1♀ (LPSNU, fig. 5A–E), Longwangping, Shengkang Town, Gucheng County, Xiangyang City, Hubei Province, China, 7.V.2023, leg. Mao-Ye; 1♀ (YZU), Hongshiyao Village, Huayang Town, Yang County, Shaanxi Province, China, 33.64°N, 107.49°E, Alt. 1270 m, 12.V.2018, leg. Xiaoqing Lu; 1♂ (CZJL), Zhuque Forest Park, Huyi District, Xi’an City, Shaanxi Province, China, Alt. 1500 m, 5.VII.2021. leg. Zi-Jun Liu; 1♀ (LPSNU ex CZJL), Shuitianping, Yangguan Village, Zhenping County, Ankang City, Shaanxi Province, China, 20.VII.2023, leg. Zi-Jun Liu.

#### Description of female.

Similar to male, but body length: 12.27–16.2 mm (3 specimens). One of the specimens (Fig. 5A–D), body length: 16.2 mm, antennae 1.2 times as long as body, length (mm) of each antennomere: scape = 3.25, pedicel= 0.5, III = 3.6, IV= 2.5, V = 2.0, VI = 1.75, VII = 1.5, VIII = 1.4, IX = 1.25, X = 1.0, X I = 0.75; elytra 1.72 times as long as wide.

#### Comments.

According to Mr Mao Ye (pers. comm.) an unknown rotten vine was broken apart by hand, exposing numerous ants, a live male adult (Fig. 4E–F) and a female pupa (Fig. 5E) of *E.ziranzhiyi.* The live adult was placed on surface litter and the pupa was placed on a dried leaf for photographs. The pupa eclosed after several days.

The two inward oblique and sub-oval pubescent pink spots on the vertex are not well-defined on some males (Figs 3A, 4A), the two sub-rounded pink spots on the sides of the center of the frons are not well-defined on some males (Fig. 3 in Yamasako and Lin 2016, fig. 4D) and a female deposited in YZU.

#### Distribution.

China (Hubei, Shaanxi).

##### ﻿Key to species of *Eurymesosa*

**Table:** 

1	Vertex covered with two inward oblique and sub-oval pink pubescent spots (Figs 3A, 3C, 3E, 4A, 4D, 5A, 5D) close to upper lobe of eyes	***E.ziranzhiyi***
–	Vertex covered with two longitudinal and wide pubescent bands (Figs 1A, 1D, 1F, 1I, 2A, 2D, 2F, 2I, 2M) close to upper lobe of eyes	**2**
2	Pubescent bands on vertex brown with light pink border (Fig. 1A, 1D, 1F, 1I). Each elytron covered with patchy dark brown maculae in basal half, with a large irregular black macula behind middle; disc with patchy dark brown maculae in middle and near apical 1/4 (Fig. 1A, 1F). Femora, apical 2/3 of tibiae and dorsum of two basal joints and last joint of tarsi covered with short light pink pubescence (Fig. 1A–C, F–H)	***E.allapsa***
–	Pubescent bands on vertex yellowish brown (Fig. 2A, 2D, 2F, 2I, 2M). Each elytron covered with patchy yellowish-brown maculae in basal half, with several small irregular black maculae behind middle; disc with patchy yellowish-brown maculae in middle and near apical 1/4 (Fig. 2A, 2F, 2I, 2M). Femora, apical 2/3 of tibiae and dorsum of two basal joints and last joint of tarsi covered with short yellowish-brown pubescence (Fig. 2A–C, 2F, 2G, 2I–K, 2M–O)	***E.ventralis***

## Supplementary Material

XML Treatment for Eurymesosa

XML Treatment for Eurymesosa allapsa

XML Treatment for Eurymesosa ventralis

XML Treatment for Eurymesosa ziranzhiyi

## ﻿Additional information

### Author ORCIDs

Gui-Qiang Huang https://orcid.org/0000-0002-0063-8157

Ling-Rui Xu https://orcid.org/0009-0002-4883-1025

Xian Zhou https://orcid.org/0009-0008-0108-5292

Gui-Mei Zhang https://orcid.org/0000-0002-4969-3288

### Data availability

All of the data that support the findings of this study are available in the main text.
